# The Benefits and Risks of Certain Dietary Carotenoids that Exhibit both Anti- and Pro-Oxidative Mechanisms—A Comprehensive Review

**DOI:** 10.3390/antiox9030264

**Published:** 2020-03-23

**Authors:** Homer S. Black, Fritz Boehm, Ruth Edge, T. George Truscott

**Affiliations:** 1 Department of Dermatology, Baylor College of Medicine, Houston, TX 77030, USA; 2 Photobiology Research, Internationales Handelszentrum (IHZ), Friedrichstraße 95, 10117 Berlin, Germany; fritz.boehm@gmx.de; 3 Dalton Cumbrian Facility, Westlakes Science Park, The University of Manchester, Cumbria CA24 3HA, UK; 4 School of Chemical and Physical Sciences, Keele University, Keele, Staffordshire ST5 5BG, UK; t.g.truscott@keele.ac.uk

**Keywords:** carotenes, cancer, porphyria, macular degeneration, singlet oxygen, radical reactions, β-carotene, lycopene

## Abstract

Carotenoid pigments, particularly β-carotene and lycopene, are consumed in human foodstuffs and play a vital role in maintaining health. β-carotene is known to quench singlet oxygen and can have strong antioxidant activity. As such, it was proposed that β-carotene might reduce the risk of cancer. Epidemiological studies found inverse relationships between cancer risk and β-carotene intake or blood levels. However, clinical trials failed to support those findings and β-carotene supplementation actually increased lung cancer incidence in male smokers. Early experimental animal studies found dietary β-carotene inhibited UV-induced skin cancers. Later studies found that β-carotene supplementation exacerbated UV-carcinogenic expression. The discrepancies of these results were related to the type of diet the animals consumed. Lycopene has been associated with reduced risk of lethal stage prostate cancer. Other carotenoids, e.g., lutein and zeaxanthin, play a vital role in visual health. Numerous studies of molecular mechanisms to explain the carotenoids’ mode of action have centered on singlet oxygen, as well as radical reactions. In cellular systems, singlet oxygen quenching by carotenoids has been reported but is more complex than in organic solvents. In dietary β-carotene supplement studies, damaging pro-oxidant reactivity can also arise. Reasons for this switch are likely due to the properties of the carotenoid radicals themselves. Understanding singlet oxygen reactions and the anti-/pro-oxidant roles of carotenoids are of importance to photosynthesis, vision and cancer.

## 1. Introduction

**Figure 1:**
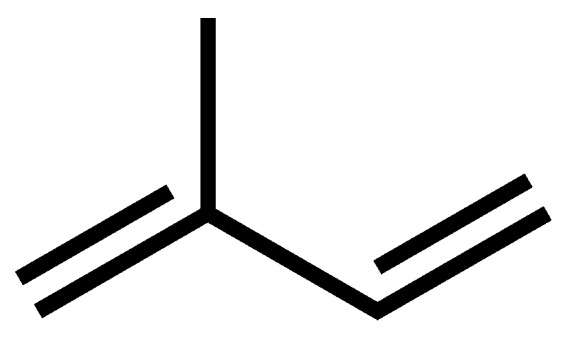
Isoprene unit.

Carotenoids are profuse throughout both plant and animal kingdoms although, in plants, their presence is often masked by chlorophyll. These pigments are widely distributed as naturally occurring constituents of fruits and vegetables, as well as causing the coloration of a wide variety of flowers, birds and marine animals. Over 600, now estimated by some to be around 750, carotenoids have been identified with about 100 finding their way into foodstuffs consumed by humans [1]. As well as fruit and vegetable intake, they are often added to human foodstuffs in order to achieve acceptable food coloration as they strongly absorb light in the region of 400–500 nm and are colored red, orange, and yellow. Chemically these pigments are tetraterpenoid, C40 compounds, consisting of eight isoprenoid residues (Figure 1).

**Figure 2:**
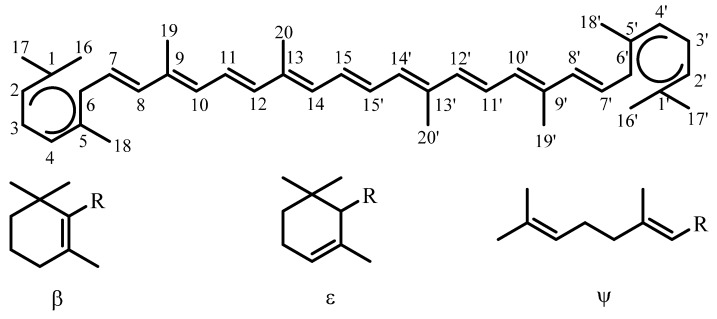
The numbering system of the stem name ‘carotene’ and three of the seven prefixes which designate the end groups found in natural carotenoids.

The central unit normally contains 22 carbon atoms, with nine double bonds and four side-chain methyl groups, but rearrangements of this are still classified as carotenoids if the two central methyl groups are retained. The end groups are often cyclized, and do not have to be identical. They are each affixed a Greek letter name (Figure 2) and, when the end groups are not identical, the Greek letter prefixes are given in alphabetical order, with the first end group having the unprimed numbers in the numbering system of the hydrocarbon chain (Figure 2). Although the numbering system provides a means of naming a carotenoid so that its structure can be identified, the most common carotenoids are typically given commonplace names.

β-Carotene is the orange pigment in carrots, from where this class of pigments gets its name; it is the carotenoid which is the most well-known and considered to be the ‘parent’ carotenoid. All carotenoids which contain oxygen moieties are collectively termed xanthophylls. These may contain, for example, hydroxyl, methoxyl, aldehyde or carboxylic acids, most commonly in the 1–6 or 1’–6’ ring positions.

Carotenoids which have over 40 carbon atoms are known as homo-carotenoids, while those with fewer than 40 are termed apo-carotenoids when carbon atoms have been removed from the end of the molecule, or nor-carotenoids when they have been lost from within the chain. The general name of retro-carotenoid is given to those where the single and double bonds within the conjugated chain have been shifted by one position.

**Figure 3:**
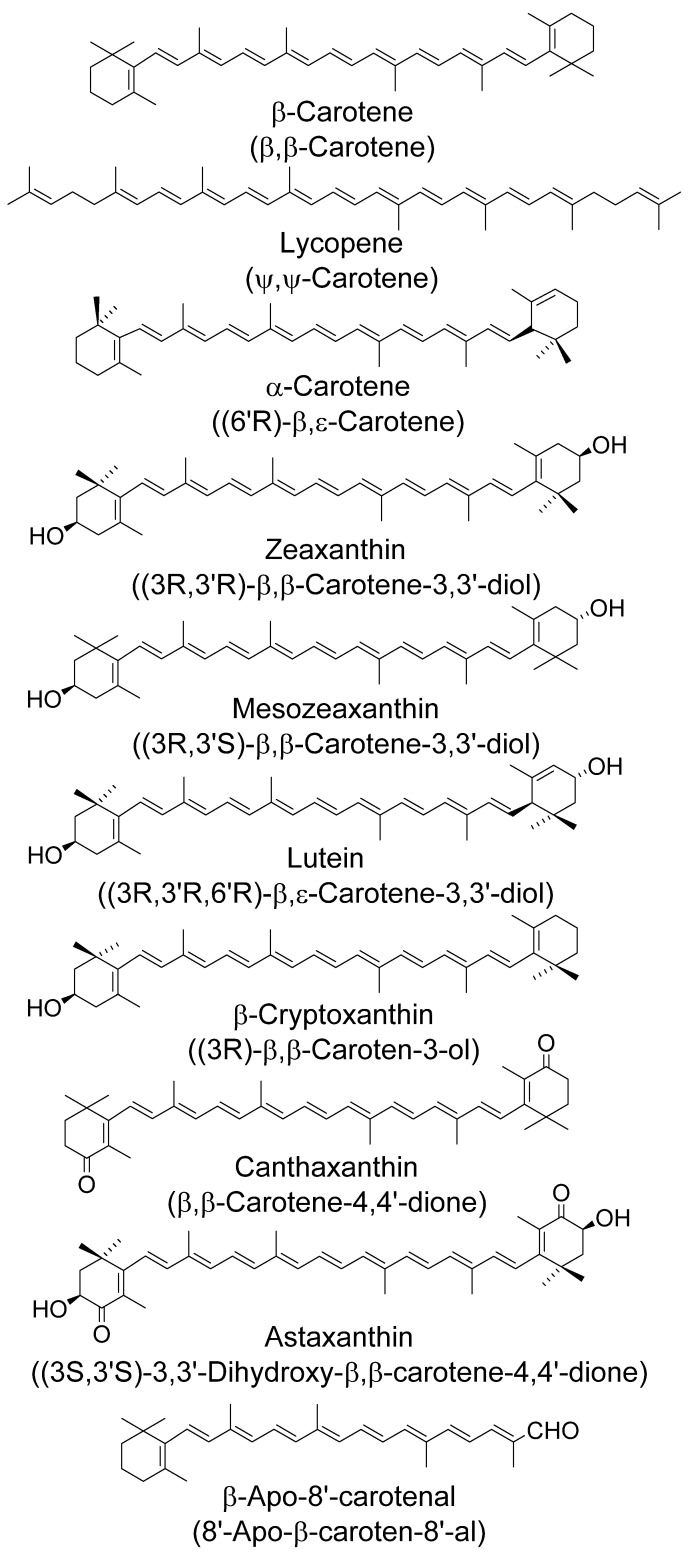
Structures of all-trans carotenoids and analogues.

The majority of carotenoids found in nature are in their all-trans (all-E) forms, with only a few exceptions, such as those in photosynthetic reaction centers. Chirality can also be observed within carotenoids, with the C-3 of the 3-hydroxy-β-ring being the most common chiral center, as seen for the stereoisomers zeaxanthin and meso-zeaxanthin. Their structures, and those of the various carotenoids discussed in this review, are given in Figure 3.

Most carotenoids are insoluble in water but dissolve readily in hydrocarbon solvents, such as benzene and hexane. The xanthophylls also dissolve in alcohols, but hydrocarbon carotenoids only dissolve to a small extent in such solvents. Of course, the shape and size of a carotenoid will affect its ability to fit into cellular membrane structures. All-trans isomers, which are rigid linear molecules, aggregate more readily than cis isomers [2]. The nature of the end groups is also of importance; for example, carotenoids containing polar groups can interact with molecules at the water–membrane interface, e.g., lutein and zeaxanthin. In some cases, they can also span the membrane, thus, increasing membrane rigidity and mechanical strength, e.g., zeaxanthin [3].

While carotenoids are eaten in significant quantities in foodstuffs present in a normal diet, more recently, they are also consumed via food supplementation. A major reason for this may well be due to claims that they offer significant health benefits; however, there are also counter claims that they can be damaging to human health. This ‘switch’ may well be linked to anti- and pro-oxidation processes involving carotenoids and, in this review, we discuss in vitro, ex vivo, animal and human studies. Carotenoids are considered to be antioxidants due to their ability to quench many reactions that lead to lipid peroxidation.

The beneficial and deleterious roles on human health of the dietary carotenoids may be due to several factors, but antioxidant and pro-oxidant processes seem to be particularly important. Other factors not discussed in this review include the effects of carotenoids on gap junction potentials [4] and insulin-like growth factors [5]. The antioxidant behavior concerns two distinct oxidizing species (i) singlet oxygen—sometimes called ‘active’ oxygen and (ii) free radicals of which there are several different types (discussed below). The pro-oxidative, deleterious effects are also believed to involve free radicals—often where the carotenoid itself behaves as an oxidizing radical. In some processes, high oxygen tension (above 150 Torr, the O_2_ pressure in normal air) has been shown to lead to a switch to such pro-oxidative damage.

Carotenoids serve in a protective role against photosensitization, generating singlet oxygen via endogenous photosensitizers such as porphyrins. For example, β-carotene is an important micronutrient that effectively prevents lethal hematoporphyrin photosensitization in mice and is employed in the treatment of protoporphyrin-induced photosensitivity in the human genetic disease erythropoietic protoporphyria (EPP) [6,7]. However, its role (and that of other dietary carotenoids) in affecting a reduction in other serious diseases, for example, cancer and heart disease, are in question [8,9].

In this review we attempt to link the clinical, in vivo and ex vivo results with the underlying molecular mechanisms obtained from in vitro studies, with an emphasis on antioxidative processes and the possible switch to damaging pro-oxidation.

## 2. Epidemiological Studies and Clinical Trials

### 2.1. Porphyria

The intermediates in the formation of haem are porphyrins (natural tetra pyrrole metabolites). Haem is composed of an iron atom in the ferrous state, chelated to a specific tetrapyrrole called protoporphyin IX. Enzymic dysfunction can lead to a group of diseases called porphyria. These diseases can be acquired or inherited. When protoporphyrin IX (PPIX) builds up, the consequence is severe skin photosensitivity [10]. Other porphyrins are associated with different porphyria. Thus, when uroporphyrin I (UPI) builds up, in the urine and plasma [10], the result is the disease *porphyria cutanea tarda* (PCT) causing both photosensitivity and actinic elastosis. On exposure to light, the porphyrins are converted to their triplet excited states and these, in turn, transfer their excess energy to molecular oxygen, resulting in the formation of singlet oxygen (^1^O_2_) which causes the photosensitivity. As well as ^1^O_2_, other reactive oxygen species (ROS) can be formed and these include peroxyl radicals, hydroxy radicals, the superoxide radical anion and hydrogen peroxide, and these can also lead to cell membrane damage [10,11,12], which is discussed further below. β-carotene is widely used to treat the skin photosensitivity which arises in EPP [13,14,15] but it is much less useful in ameliorating the photosensitivity associated with PCT [7]. However, it is possible that other carotenoids may be more effective and that combinations of antioxidants may enhance the protective effects.

β-Carotene has been used for over 30 years to ameliorate the skin photosensitivity arising from excess protoporphyrin. In the early studies of Mathews-Roth and co-workers [7], about 84% of those with EPP benefited from large daily doses of β-carotene. Currently the normal dose used is 75–150 mg/day. The molecular mechanisms associated with such photosensitivity are discussed below.

PCT has also been considered for treatment with β-carotene. Unlike PPIX, uroporphyrin is soluble in water. (Interestingly, it is this disease which is associated with the myths concerning vampires!). β-carotene does not appear to protect against the photosensitivity in PCT; however, it may be that combinations of antioxidants, with or without β-carotene, may enhance skin protection [16]. These investigators used various cell lines and human cell cultures and compared PPIX and UPI as photosensitizers. While both β-carotene and lycopene gave significant protection for PPIX photosensitization, neither protected against UPI photosensitization. However, a triple combination of β-carotene plus vitamin C and E (α-tocopherol) did give significant protection against UPI photosensitization, approximately the same protection as a triple combination with PPIX. The vitamin C and E alone gave no protection and while no molecular mechanism has been published for this finding, a possible role of vitamin C with UPI is discussed below.

β-carotene is also used to treat other diseases associated with UV, such as solar urticaria, polymorphic light eruptions (PLE), and photoallergic drug reactions, although in some cases the outcome seems variable. Mathews-Roth [7] found only 33% benefited from β-carotene against PLE and only 20% for other forms of UVB-induced photosensitivity. However, other researchers [17] claim significant benefits for β-carotene, with over 50 of the 66 patients studied reporting significantly increased tolerance to sunlight. The detailed molecular mechanisms of PLE/β-carotene are not understood—the UV absorbing chromophores are not established, as there is no excess porphyrin for these diseases. Of course, several possibilities exist for UVB absorbers, including the amino acid moieties tryptophan, tyrosine and cysteine, as well as flavins, etc. A range of ROS may then be formed and at least some of these could be ameliorated by carotenoids, such as β-carotene.

### 2.2. Cancer

A seminal study, in 1981, reported that individuals that consumed a greater amount of green leafy and yellow vegetables exhibited a lower risk for cancer [18]. Aware that these types of vegetables were rich in carotenoids, particularly β-carotene, and aware of the latter’s singlet oxygen quenching and antioxidant capacity, the authors suggested that β-carotene might be responsible for the anti-cancer effect observed. Later, that same year, a report of a 19-year longitudinal case-control study concluded that smokers had a much greater incidence of lung cancer than non-smokers and that the greatest incidence occurred in the quartile that reflected the lowest β-carotene intake [19]. Almost all of a large number of prospective and retrospective epidemiological studies of either the intake of foods rich in β-carotene or high blood levels of β-carotene reported a strong association with reduced risks of various kinds of cancers including lung, skin, colon, breast, and prostate [1,20]. For example, almost all of nine major case-controlled studies of lung cancer found a lower risk for persons with a higher intake of carotenoids [1]. Furthermore, significant inverse associations of lung cancer with blood (plasma or serum) β-carotene concentrations were found in seven nested case-controlled studies [1]. The incidence of non-melanoma skin cancer (NMSC) was also found to be inversely associated with serum β-carotene levels in a case-control study [21]. It is with skin and lung cancer that these associations have been the strongest, whereas associations of dietary intake of β-carotene and other cancers have largely been inconclusive. One reason for this is that in epidemiological studies, such as in case-control design, data for intake of fruits and vegetables may suffer from patient dietary recall and selection bias.

The effects of carotenoids, mainly β-carotene and lycopene, on a range of cancer biomarkers have been reported. These include those for lung cancer [22,23], colon cancer [24] and breast cancer [25,26]. The outcome frequently depends on a range of factors, such as cell type. A particularly important biomarker for prostate cancer is the Protein Specific Antigen (PSA), and this is discussed in some detail below. For many of the cancers, the results of the biomarker studies suggest beneficial effects of carotenoids—but not in some specific cases (as discussed below for lung cancer, β-carotene increases lung cancer in heavy smokers).

#### 2.2.1. Breast Cancer

A pooled analysis of cohort studies found non-significant associations for total fruit, total vegetables, and total fruit and vegetable intake and breast cancer [27]. In this regard, a systematic review and meta-analysis of prospective studies of dietary intake and blood concentrations of six dietary carotenoids found that blood concentrations of carotenoids are more strongly associated with reduced breast cancer risk than carotenoids assessed by dietary questionnaires [28]. A longitudinal study, in which serum concentrations of β-carotene, α-carotene, β-cryptoxanthin, lycopene, and lutein plus zeaxanthin were measured at baseline, and repeated across time, indicated that α- and β-carotene were inversely associated with breast cancer in postmenopausal women [29]. A positive association was observed for lycopene, although the sample size was small. Contrarily, an epidemiologic cohort study based on blood carotenoid intake as a biomarker for consumption of fruits and vegetables found no significant associations between breast cancer risk and serum carotenoids in postmenopausal women [30]. More recently, a nested case control study of plasma carotenoids and breast cancer risk, over 20 years of follow-up in the Nurses’ Health Study, found that higher concentrations of α-carotene, β-carotene, lycopene and total carotenoids were associated with an 18–28% statistically significant lower risk of breast cancer [31]. This was particularly the case for more aggressive and fatal disease. It is difficult to draw clear conclusions about the role of carotenoids in breast cancer incidence from the many conflicting studies. Nevertheless, studies of subgroups of pre- and postmenopausal women may provide further insight into the role of specific carotenoids in this disease.

#### 2.2.2. Prostate Cancer

As noted previously, dietary factors have been implicated as risk factors for prostate cancer [32]. Studies into the role of carotenoids in reducing prostate cancer risk have been as contradictory and inconclusive as with other cancers. The first epidemiological study (case-control) to demonstrate a reduced risk of prostate cancer with increased intake of β-carotene was initiated in 1981 [33]. A number of studies followed that supported an association of β-carotene with reduced prostate cancer risk, whereas others did not [34]. As individual carotenoid concentrations began to be investigated with regard to cancer risk, lycopene became the carotenoid of interest. A study involving 47,365 persons, the so-called Health Professionals Follow-Up Study, examined the role of lycopene upon prostate cancer risk through the evaluation of tomato products. Participants with high tomato sauce (a strong predictor of plasma lycopene) and lycopene intake were at reduced risk of prostate cancer [35]. A pooled analysis of 15 studies involving 11,239 cases of prostate cancer and 18,541 controls examined associations of the concentrations of seven carotenoids and risk of prostate cancer and associations by stage or grade of disease [36]. None of the carotenoids tested, including lycopene, were associated with an overall risk of prostate cancer. However, lycopene exhibited a statistically significant heterogeneity related to the stage of the disease, i.e., the risk of aggressive disease was inversely associated with lycopene. A phase II randomized and controlled clinical trial was conducted to evaluate the safety and effect of several doses of lycopene to men with clinically localized prostate cancer and to assess the impact on intermediate endpoints [37]. Serum-free testosterone decreased and total estradiol increased significantly in those patients receiving 30 mg lycopene daily, while no significant increases in serum prostate specific antigen (PSA) occurred, although these changes were not significant compared to the control arm of the study. Nevertheless, the authors suggested that mechanisms other than antioxidant activity, perhaps mediated by steroid hormones, might also be responsible for lycopene’s beneficial effect. A meta-analysis of 34 observational studies (10 cohort, 11 nested case-control and 13 case-control studies) reported that both α-carotene and lycopene were inversely associated with the risk of prostate cancer, although neither could lower the risk of advanced prostate cancer [38], while β-carotene intake or its blood levels were not associated with a lower prostate cancer risk. A randomized double-blind placebo-controlled phase I-II study examined the effects of dietary supplements, including lycopene, in men with multifocal high grade prostatic intraepithelial neoplasia and/or atypical small acinar proliferations (precancerous prostatic lesions) [39]. The investigators found that the administration of high doses (35 mg) of lycopene was associated with a higher incidence of prostatic cancer at re-biopsy and recommended that this supplement should be avoided. However, in updated results, Giovannucci et al. [35] have shown that lycopene is associated with a reduced risk of lethal prostate cancer rather than indolent prostate cancer. Indeed, this may explain the variation in outcomes discussed above. Presumably, lethal prostate cancer is the preferred endpoint, rather than total prostate cancer, for epidemiological studies into the value of lycopene against prostate cancer [36].

Indeed, several small clinical trials have shown benefits against prostate cancer for lycopene, employing typical doses around 15 mg/day. One such trial [40] was based on PSA measurement over one year in a group of patients, all of whom had established prostate cancer, and suggested that over 60% benefited from the reduced rate of acceleration of PSA or, in about 20% of the cases, an actual reduction in PSA. In another trial, patients who were scheduled to undergo radical prostatectomy received supplementation with lycopene or did not [41]. It was found that the plasma PSA level decreased by 18% in the intervention group, while it increased by 14% in the control group. They also reported that, following the prostatectomy, about twice as many men in the lycopene group had tumors of 4cc or less, compared to the control group. These researchers point out that carotenoids (such as lycopene and β-carotene) may modulate processes related to mutagenesis, carcinogenesis, cell differentiation and proliferation independently of their role as antioxidants, one such role being to increase gap junction intercellular communication.

Of course, as noted above, intake of dietary antioxidants other than lycopene may also contribute to benefits against prostate cancer [33]. More recently, Vance and co-workers [42] have demonstrated that the intake of antioxidants was associated with less oxidative stress among men with incident prostate cancer. Their results indicate additional research on the relationship between dietary antioxidants and prostate tissue redox status and carcinogenesis is needed to confirm if such a relationship may influence disease severity, progression and recurrence.

#### 2.2.3. Colon Cancer

Fruit, vegetables and, by association, carotenoid intake have also been implicated in the prevention of colon cancer [43]. Only half of the six studies reviewed reported that the combined consumption of vegetables and fruit reduced colorectal cancer risk. Because of these inconsistencies, a Canadian study (case-control) was undertaken to examine the association between dietary carotenoids and colon cancer risk [44]. Overall, no association was found between dietary intake of carotenoids and colon cancer risk. However, among never-smokers, a significant reduced risk was associated with intake of β-carotene. An inverse association was found between lycopene intake and colon cancer risk among smokers. In a multi-ethnic cohort study, using a quantitative food frequency questionnaire and carotenoid densities, the researchers reported no significant associations between intake of individual and total carotenoids and colorectal cancer risk in either men or women, depending upon the model employed in analysis (basic or multivariate [45]). β-cryptoxanthin showed a small protective effect in men. Lycopene intake was related to an increased risk of rectal cancer in men. Total β-carotene decreased the risk of colorectal cancer in male smokers (this is an interesting observation considering what has been reported in lung cancer). A Chinese case-control study measured serum levels of α-carotene, β-carotene, β-cryptoxanthin, lycopene and lutein/zeaxanthin and found that colorectal cancer incidence was associated with lower levels of α-carotene, β-cryptoxanthin and lycopene among the specific Chinese population [46]. Thus, the role of carotenoids in the risk of colorectal cancer is as inconclusive as with other cancers.

#### 2.2.4. Skin Cancer

It was assumed that Non-Melanoma Skin Cancer (NMSC) risk could be ameliorated by β-carotene based upon its known strong singlet oxygen quenching and antioxidant properties. A case-control study found that the incidence of NMSC was inversely related to the level of serum β-carotene [21]. However, in a controlled clinical trial in which 50 mg of oral β-carotene was administered daily, there were no significant differences in any of the primary endpoints after five years [8]. The amount of β-carotene administered in this study increased the plasma carotenoid levels nearly 10-fold above that of controls. A second randomized trial in Australia examined the influence of 30 mg/day supplementation of β-carotene on the incidence of basal and squamous cell carcinomas over a four-year period [47]. A small, but statistically insignificant, increase (1508 vs 1146/100,000; 1.315 (0.84–2.19) in the incidence of squamous cell carcinoma was observed with β-carotene supplementation. The investigators concluded that there were no beneficial or detrimental effects on skin cancer rates as a result of β-carotene supplementation. Further evaluations from nested case-control studies showed that β-carotene supplementation had no effect on any of the controlled patient subgroups, i.e., numbers of previous skin cancers, age, gender, smoking, skin type or baseline plasma β-carotene levels [48,49]. Interestingly, those persons who were in the highest quartile of the initial plasma β-carotene level had the lowest risk of death from all causes, but β-carotene supplementation did not affect mortality.

Few studies have examined the influence of β-carotene on the occurrence of melanoma skin cancer. Nevertheless, in three case-control studies, no association was found between blood carotenoid levels and the risk of melanoma [50,51,52]. In one of these studies the risk of melanoma among men and women in the three highest quartiles of β-carotene intake actually increased by 40–50%, albeit neither the risk for the fourth quartile nor the test for trend was statistically significant [52]. A side note of interest is that β-carotene supplementation (50 mg/day for 36 months) has been reported to have beneficial effects in dysplastic nevus syndrome, producing a statistically significant retardation in the size of dysplastic nevi on some body sites. β-Carotene did not have a significant effect on the average number of newly developed nevi [53]. A meta-analysis of randomized controlled trials found that β-carotene supplementation had no effect on the incidence of melanoma (RR, 098; 95% CI, 0.65–1.46) nor upon NMSC risk (RR, 0.99; 95% CI, 0.93–1.05) [54].

#### 2.2.5. Lung Cancer

As noted above, a 1981 19-year longitudinal case-control study found that smokers had a markedly greater incidence of lung cancer, compared to non-smokers, and that the greatest incidence occurred in the quartile that exhibited the lowest carotene intake [19]. Furthermore, of nine major case-control studies of lung cancer and carotene intake, all showed a lower risk of lung cancer among persons with a higher intake of carotenoids [1]. In addition, seven nested case-control studies of lung cancer and blood (plasma or serum) β-carotene levels found significant inverse associations when controlled for tobacco use and other confounding factors. It is interesting to note that the majority of these studies found stronger inverse trends with fruit and vegetable intake than that with carotenoid intake, providing a hint that the combination of β-carotene with other micronutrients may have had a greater effect than the carotenoid alone. Thus, almost all of the large number of prospective and retrospective epidemiological studies of either the intake of foods rich in β-carotene, or high levels of blood β-carotene, reported a strong association with reduced risks of lung cancer [55]. With overwhelming epidemiological evidence for the cancer-preventive effect of β-carotene, it was both surprising and disturbing when results from the eight-year intervention trial of the Alpha-Tocopherol, Beta-Carotene Cancer Prevention Study (known as the ATBC trial) reported an 18% increase in the incidence of lung cancer in male smokers who were administered 20 mg/day of a β-carotene supplement, compared to non-supplemented controls [56]. Previously, five intervention trials had been undertaken. Except for a trial in which β-carotene had been co-administered with selenium and α-tocopherol, none of these provided evidence of a protective effect of β-carotene [1]. A second lung cancer chemoprevention trial, the β-carotene and retinol efficacy trial (known as CARET), sought to determine whether the combination of β-carotene, with its presumed antioxidant effects, and the tumor-suppressing molecular actions of vitamin A could reduce the incidence of lung cancer in high risk populations [57]. Participants in the intervention arm of the study received 30 mg/day of β-carotene plus 25,000 IU daily of retinol. β-carotene plus retinol was associated with an increased incidence of lung cancer among the entire cohort of 18,314 smokers, former smokers and asbestos-exposed participants (RR, 1.28; 95% CI, 1.04–1.57; *p* = 0.02). In view of the ATBC trial results and in consideration of the safety of participants in the CARET trial, the intervention was halted about 21 months early. This pretty much signaled the end of β-carotene clinical trials. However, as discussed above, very high doses (75–150 mg/day) of β-carotene continue to be used to treat skin photosensitivity. This is discussed by Bayerl [58] who points out both the criticisms of the ATBC and CARET trials and also suggests that the use of β-carotene should be discussed with patients only after weighing the risk/benefit ratio.

### 2.3. Age-Related Macular Degeneration (AMD)

AMD is the leading cause of blindness in the Western world. The carotenoid (xanthophyll) pigments lutein and zeaxanthin accumulate in the macula (zeaxanthin is in two stereo isomeric forms, one of which is called meso-zeaxanthin—the structures are given in Figure 3). AMD may be associated with low levels of lutein and zeaxanthin in the diet, serum or retina, and exposure to blue light. The area on the retina that can perceive the finest details is known as the “yellow spot” or “macula”. It surrounds the fovea, which contains the highest density of visual cells. These molecules in the macula lutea are concentrated up to a combined concentration of about 1 mM, (ratio 1:1:1) the highest concentration of carotenoids found anywhere in the primate body [59]. Epidemiological findings over many years show how lutein, zeaxanthin and mesozeaxanthin could contribute to a risk reduction of drusen formation, neovascularisation and, especially, Age-Related Macular Degeneration based on both a ‘simple’ light filter effect and also reduced damage by quenching various forms of Reactive Oxygen Species, including both singlet oxygen and various free radicals [60].

At least two roles for the macular pigments are likely—a simple light-filter effect, and an antioxidant effect to minimize the damage due to ^1^O_2_ and other ROS (the macula has a high metabolic rate and, as a consequence, a high oxygen requirement). While several roles may be important, in this review, we concentrate on the anti/pro-oxidation processes involving the macular carotenoids. Some proposed mechanisms concerning such processes are given below, suggesting distinct roles for lutein, zeaxanthin and also for lycopene (even though lycopene does not accumulate in the macula).

## 3. Animal Studies

As noted earlier, β-carotene was shown to prevent lethal hematoporphyrin photosensitization in mice and is employed in the treatment of protoporphyrin-induced photosensitivity in the human genetic disease erythropoietic protoporphyria (EPP) [6,7]. In addition to their original investigations of EPP, Mathews-Roth et al. [13,14,61] showed that β-carotene exerted a small, but statistically significant, increase in the minimal erythema dose (MED) of sunburn in man. These studies were extended to show a similar protective effect with phytoene in guinea pigs [62]. Phytoene is the triene precursor to β-carotene and absorbs strongly in the UVB range of the electromagnetic spectrum.

Based upon these observations, Epstein examined the potential influence of intraperitoneally injected β-carotene on UV-induced tumor formation in the hairless mouse [63]. The carotenoid was found to exert a limited, but protective, effect with regard to time of tumor appearance and tumor growth. In mixed chemical/UV carcinogenic protocols, oral administration of β-carotene or canthaxanthin was found to delay the appearance of skin tumors [64]. Employing a UV-carcinogenic protocol and the Skh-Hr-1 hairless mouse model, it was determined that canthaxanthin, but not β-carotene or phytoene, could slow the development of subsequent tumors if administered immediately after development of the primary tumor, indicating a promotion stage effect [65]. Nevertheless, phytoene, when administered (injected intraperitoneally) for ten weeks prior to exposure to a single, large, tumor-initiating fluence of UV was photoprotective. Additional studies confirmed that oral (dietary) administration of 0.1% (w/w) of either β-carotene or canthaxanthin, for six weeks prior to exposure to a single, tumor-initiating UV dose, and then continuing carotenoid administration during the progression stage of carcinogenesis, could significantly prevent the development of skin tumors [66]. β-carotene, when administered only before UV exposure, provided no significant protection. Thus, in this study, it was inferred that photoprotection resulted only when the carotenoid was administered during the progression phase of tumor development [67]. Subsequently, in a somewhat complicated protocol (complicated from the standpoint that animals received the varying levels of carotenoids in different “run-in” feeding periods ranging from 4 days to one month and that animal ages at the time of irradiation ranged from 8 to 20 weeks), the investigators found that both canthaxanthin and β-carotene provided significant photoprotection to carcinogenesis when the pigment was administered throughout the course of the study and at a level of 0.07% (w/w) in the diet [68]. Lower levels did not provide significant protection. Employing the C3H/HeN mouse model in an UV-carcinogenic protocol, a diet supplemented with 1% (w/w) canthaxanthin significantly reduced the average skin tumor burden, although the tumor latent period was unaffected [69,70].

At this point, there seemed to be overwhelming experimental evidence to substantiate the anti-UV-carcinogenic potential of β-carotene. However, in 1998, a laboratory study employing a semi-defined diet, compared to the earlier studies employing commercial closed-formula diets in which photoprotection was observed, not only failed to provide protection against UV-carcinogenesis, but resulted in significant exacerbation by β-carotene and astaxanthin, but not by lycopene [71].

Matrix analyses of experimental variables in those studies showing photo-protection or exacerbation of carcinogenesis indicated that animal age, carotenoid dose, or type of diet were the most probable causes of the divergent responses to β-carotene. No significant differences in the tumor latent period occurred in young animals (2.5 months of age) fed the semi-defined diet, regardless of β-carotene dose (either 0.07% or 0.3% (w/w) of diet)) Nevertheless, tumor multiplicity was significantly increased at each higher dose of the carotenoid. Both young and older animals were equally responsive to exacerbation of UV-carcinogenesis with respect to this tumor parameter [72]. Thus, animal age was not an adequate determinant of carcinogenic response to β-carotene.

It seemed logical that the presence and interactions with other dietary factors in the closed-formula diet, and the lack thereof in the semi-defined diet, might be responsible for the disparate influence of β-carotene on UV-carcinogenesis [73]. An explanation of the two types of rations employed in the β-carotene studies is warranted. Commercial rodent rations are examples of closed-formula diets. They are composed of feedstuffs such as milled grains, alfalfa, and so forth. The composition of these diets will vary with season, regional locale of source, and market prices. Thus, the specific composition of each lot will vary, making it impossible to quantitatively duplicate. It was this type of ration that was employed in the earlier studies in which photo- protection was observed. Semi-defined diets are those formulated from refined foodstuffs, e.g., corn starch, refined oils, casein, and defined vitamin and mineral supplements. It is this type of diet that was employed where exacerbation of UV-carcinogenesis was observed. Indeed, the influence of β-carotene-supplemented diets is reflected in Table 1 [74]. Care was taken in this study to ensure that comparable levels of the carotenoid were consumed in both diets and none of the effects, or lack thereof, on carcinogenic expression could be attributed to differential carotenoid intake [74]. Here, it is worth pointing out that the 0.07% β-carotene supplementation in the mouse is equivalent to consumption, on a body weight basis, to ~160-fold higher levels than that employed in many clinical trials (50 mg/day per ~75 kg body weight). The practical relevance of employing a higher dose of the carotenoid, even if effective as a photo-protectant, would be questionable. Nevertheless, it is apparent from these studies that dietary components, such as other carotenoids, their isomers, or some unidentified phytochemicals, are present in the closed-formula ration that are absent in the semi-defined diet and which potentiate the carcinogenic response to β-carotene. Moreover, β-carotene has been shown to exhibit either limited antioxidant protection or to behave as a pro-oxidant under oxidative stress conditions [75]. The possible molecular mechanisms and the role of oxygen concentration are discussed below. Some of these mechanisms suggest a role for the interaction of vitamin C with carotenoid radicals. There was no listed vitamin C content in the commercial closed-formula ration in those studies in which β-carotene had provided photo-protection or in those in which no effects of the carotenoid were observed [76]. Studies in which β-carotene induced exacerbation of carcinogenic expression employed the semi-defined diet to which vitamin C had been added. The same exacerbation occurred, however, when the semi-defined diet was devoid of vitamin C. In previous studies, it was proposed that either a lack of adequate levels of vitamin C in smokers or an excess of vitamin C could stoichiometrically drive the proposed repair mechanism (see reaction Equations (7)–(10) in Molecular Mechanisms, Section 4.12) to a pro-oxidant state that, in turn, would lead to an increased level of β-carotene radical cation and block the effective antioxidant activity of vitamin E. This might explain the divergent results obtained from the two diets [77].

Hairless mice were fed β-carotene-supplemented semi-defined diets containing varying levels of vitamins C and E (either increasing their concentrations or reducing them to reflect levels found in closed-formula rations) and subjected to a UV-carcinogenic protocol). The addition of high levels of vitamin E (10-fold) or vitamin C (six-fold) did not ameliorate the exacerbated effects of β-carotene on carcinogenesis, nor did the complete removal of vitamin C from the diet have an effect. However, by reducing the level of vitamin E in the semi-defined diet to that present in the closed-formula ration, there was a significant (nearly two-fold) increase of exacerbation of UV-carcinogenesis [78]. These effects are depicted in Table 2 [79].

When compared to the control, it can be seen that β-carotene supplementation of the semi-defined diet, at the lower level of vitamin E, significantly (*p* = 0.0065) increased tumor multiplicity nearly six-fold. These data suggest that β-carotene and vitamin E interact to partially meliorate the pro-carcinogenic influence of the former. The data with vitamins C and E supplementation also suggest that antioxidant supplementation has a limited range of upregulation in healthy, well-nourished individuals.

While proposed/speculated mechanisms are discussed below, it is clear that dietary factors can have a profound influence on β-carotene’s capacity to influence carcinogenic expression [74]. It has been suggested that β-carotene, so prevalent in the green and yellow vegetables and fruits (which led to the proposal that the carotenoid could reduce cancer risk), merely cloaks the potential anti-cancer agent [80]. The results from the ATBC and CARET clinical trials of β-carotene and lung cancer, along with the experimental results with the carotenoid and UV-carcinogenesis, have raised concern regarding the safety of β-carotene supplementation and the warning that it should not be used in cancer prevention in the general population [1,81].

Meanwhile, efforts continue (discussed below, in Molecular Mechanisms) to explain how β-carotene, a molecule with excellent antioxidant capacity under specific conditions and a singlet oxygen quencher, can act as a pro-carcinogen in UV-carcinogenesis and in lung cancer patients.

## 4. Molecular Mechanisms

### 4.1. Singlet Oxygen—General

Singlet oxygen is a significantly damaging species, both because it is energetic enough to react with most biological substrates and because it has an inherent long lifetime. It arises in nature mainly from substrates which absorb light, converting to their lowest triplet state (e.g., porphyrins and chlorophylls). These triplet states react with normal, ground state oxygen to generate ^1^O_2_. Other generation routes are possible, such as enzymatic processes and processes related to stress response [82,83]. Many ^1^O_2_ quenchers, such as all dietary carotenoids, also directly quench photosensitizer triplets, including porphyrins and chlorophylls, (to produce the carotenoid triplet, followed by loss of the energy as heat). Therefore, they can protect by reducing the ^1^O_2_ yield without actually quenching ^1^O_2_. However, in homogeneous solvents, such as hexane, benzene and xanthophylls that are soluble in alcohols, it is possible to adjust the concentrations to minimize this direct energy transfer and, hence, study only the quenching of the ^1^O_2_ by the so-called quencher (the carotenoid). However, in a real-life situation, this may not be so simple and could make studies of ^1^O_2_ in such model ex vivo systems less easy to understand—this is discussed further below with respect to HeLa cells.

### 4.2. Singlet Oxygen—Simple Solvents, Micelles and Liposomes

In homogeneous solvents, it is well established that most non-aggregated carotenoids are extremely efficient quenchers of ^1^O_2_. This efficiency is reduced by several factors—aggregation, isomerization and the number (n) of conjugated double bonds in the carotenoid. A good example of the effects of aggregation is shown by zeaxanthin in deuterated methanol/water mixtures [84].

**Figure 4:**
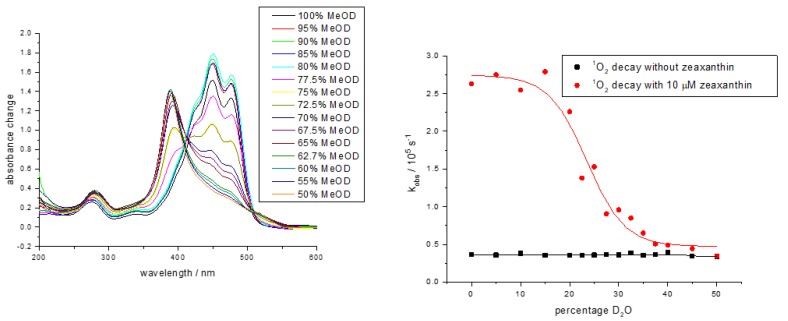
Left: ground state absorption spectra of 1 × 10^−5^ M zeaxanthin in various MeOD/D_2_O mixtures. Right: the effect of increasing D_2_O (inducing zeaxanthin aggregation) on ^1^O_2_ deactivation efficiency of zeaxanthin.

As can be seen in Figure 4, at 20% D_2_O, the quenching rate constant falls and at 37.3% D_2_O, there is effectively no quenching of ^1^O_2_; this corresponds to the water concentration, causing carotenoid aggregation. Such aggregation is also shown by Oliveros et al. [85] at the reduced rate, by an order of magnitude of ^1^O_2_ quenching by β-carotene in alcohol compared to benzene.

Studies, using benzene, have also shown cis carotenoids are less efficient at quenching ^1^O_2_ than all-trans isomers. For example, the rate constant decreases for β-carotene as the cis bond moves towards the end of the molecule from 13.5 × 10^9^ dm^3^ mol^−1^ s^−1^ for the all-trans β-carotene to 8.99 × 10^9^ dm^3^ mol^−1^ s^−1^ for the 9-cis β-carotene [86]. A time-resolved resonance Raman has an additional band—the so-called cis peak—which could affect the results from steady-state studies.

An important example of the effect of the conjugated double bond length on the ^1^O_2_ quenching, for dietary carotenoids is for lutein (*n* = 10) compared to zeaxanthin (*n* = 11). However, for α-carotene (*n* = 10), this effect is less marked. A further reduction of n (to nine, for example, as in septapreno-β-carotene) leads to a dramatic reduction in the quenching rate constant—see Table 3, below [86].

It has also been established that all β-carotene isomers share a common triplet state, twisted about the middle carbon–carbon double bond, compared to the ground state [87]. Additionally, when using hexane as a solvent, it has been demonstrated that cis isomers are formed after the reaction of the all-trans isomers with ^1^O_2_ for both β-carotene and lycopene and, furthermore, that lycopene prevents β-carotene isomerization [88]. Since the absorption spectra of the cis isomers has an additional band—the so-called cis peak—this could affect the results from steady-state studies.

Studies have also been undertaken using micelles [86,89] and liposomes [90,91,92] as cell models. The ^1^O_2_ quenching rate constants are still rather high (>10^8^ dm^3^ mol^−1^ s^−1^). In the liposome, the quenching of ^1^O_2_ did not depend on the site of the ^1^O_2_ generation (the ^1^O_2_ was generated by a water or lipid soluble photosensitizer). However, there was a significant difference in quenching by xanthophylls in liposomes compared to micelles—rate constants fell by up to 30 times in liposomes, while the quenching rate constant for β-carotene in liposomes was similar to that in neutral micellar solutions (Triton-X100). However, this seems to depend on the detergent micelle and the carotene studied. In early work β-carotene was shown not to quench ^1^O_2_ in charged sodium dodecyl sulfate (SDS) and cetyltrimethylammonium bromide (CTAB) micelles [93]. However, there was efficient quenching by xanthophylls in the charged micelles [94], while, for alpha-carotene, little quenching was observed in SDS [95].

A significant concentration effect on ^1^O_2_ quenching was also observed for xanthophylls in liposomes (especially for zeaxanthin), suggesting, as in alcohol–water mixtures, aggregation lowers quenching efficiency. Furthermore, in contrast to organic solvents, the quenching abilities of lycopene and β-carotene toward ^1^O_2_ are virtually identical in mixed micelles of Triton-X100 and Triton-X405, and also in dipalmitoylphosphatidylcholine (DPPC) liposomes. Indeed, in multi-lamellar environments, lycopene is seen to be a poorer quencher of ^1^O_2_ than β-carotene —8 × 10^9^ compared to 19 × 10^9^ dm^3^ mol^−1^ s^−1^ (unpublished—Cantrell and Truscott, 2002). A related liposome study using dimyristoylphosphatidylcholine (DMPC), reported the inhibition of ^1^O_2_-induced lipid peroxidation and showed that inhibition by β-carotene, canthaxanthin and astaxanthin was similar but that such inhibition by lycopene was significantly less (10-fold) [92]. A more recent study has also demonstrated inhibition of ^1^O_2_-induced plasma lipid oxidation by both β-carotene and fucoxanthin [95], with fucoxanthin being the better inhibitor, even though the rate constant of ^1^O_2_ quenching by fucoxanthin is lower than that of β-carotene [96].

### 4.3. Singlet Oxygen—Ex Vivo Cell Studies

Boehm and co-workers [97] used lymphocytes from human blood and water-soluble β-carotene beadlets to study the possible ex vivo quenching of ^1^O_2_. These researchers reported a significant quenching of ^1^O_2_ near to that observed in homogeneous organic solvents [98] and they suggest that this implies that these preparations lead to the β-carotene being bound at or near the cell surface, rather than embedded in the cell membrane, although, as noted above for liposomes, the ^1^O_2_ quenching by carotenoids was independent of the site of generation of ^1^O_2_ (in the region 10^8^–10^9^ dm^3^ mol^−1^ s^−1^). In a more detailed study, using similar cell models [99], four carotenoids were studied using water soluble beadlets (β-carotene, lycopene, astaxanthin and canthaxanthin). All except canthaxanthin showed a marked quenching of ^1^O_2_. These researchers also measured a cell protection factor for membrane damage by each preparation and showed the carotenoids reduced the cell damage by about a factor of two. Finally, this work also studied the effect of β-carotene taken orally rather than via incubation as beadlets. Once again, ^1^O_2_ quenching was observed at β-carotene concentrations which may be used in everyday supplementation. However, the effect was somewhat less marked than with the water soluble beadlets. Boehm and co-workers [100] also reported a protection factor for lycopene taken from the diet (from boiled tomato juice) of over six.

**Figure 5:**
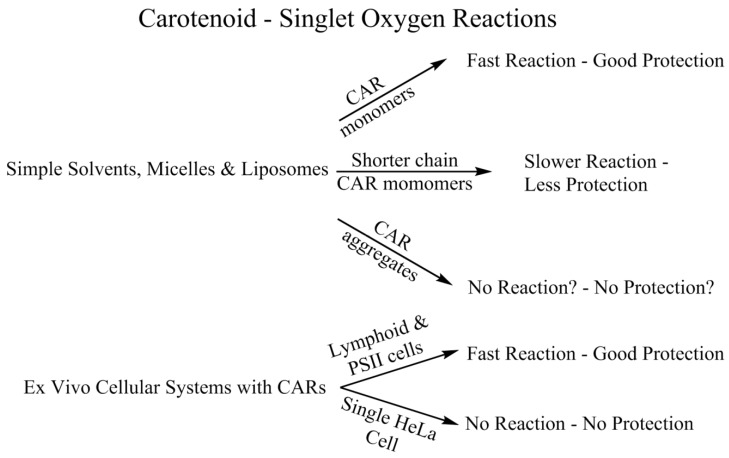
Summary scheme of carotenoid reactions with singlet oxygen.

The ability of β-carotene to quench ^1^O_2_ in cellular environments has been questioned by Ogilby and co-workers [101]. Using individual HeLa mammalian cells and microscope-based time resolved 1275 nm luminescence, these researchers could observe no quenching of the ^1^O_2_ by β-carotene (these researchers gave careful consideration to concentrations and rates of reaction in viscous intracellular environments). However, as they comment, such whole-cell systems are complex. Indeed, in a totally different cellular system, related to photosynthesis studies, Telfer and co-workers [102] used time-resolved direct detection of ^1^O_2_ at 1270 nm and steady state methods to demonstrate the formation of ^1^O_2_ in isolated Photosystem II (PSII) reaction centers and other researchers [103] have suggested quenching of such ^1^O_2_ by β-carotene. These, and related studies, have been reviewed more recently by Telfer [104]. This review addresses the roles of β-carotene bound to the PSII reaction centers where it is well known that ^1^O_2_ arises. By using chemical techniques to vary the concentration of the carotenoids, Telfer and co-workers [105] show that carotenoids quench some of the ^1^O_2_ but not all, due to the distance of the carotenoids from the source of the ^1^O_2_. This work in PSII systems suggest that, in a specific biological environment, quenching will only be efficient when the carotenoid is sufficiently close to the source of the ^1^O_2_. Clearly, there is more to learn about the interaction of β-carotene with ^1^O_2_ in an ex vivo situation. See Figure 5 for an overview of carotenoid-^1^O_2_ reactions.

### 4.4. Singlet Oxygen—Porphyria

Two processes are believed to be associated with the observed protection against porphyria by β-carotene. One is just ‘simple’ light absorption by the β-carotene—i.e., an ‘inner filter’ effect—and the second is that the ^1^O_2_ and/or porphyrin triplet state, formed upon light absorption, is efficiently quenched by the β-carotene. Within this second process, whichever quenching mechanism predominates will depend on the site-specific concentrations of oxygen and β-carotene. Clearly, the nearer the skin surface, the higher the oxygen concentration, thus, ^1^O_2_ quenching will likely predominate with direct triplet quenching being less important. PPIX has been detected in both the dermis and epidermis [106] and during both the quenching processes discussed here. All-trans β-carotene is converted to a cis–trans mixture, as has been observed previously and discussed above [87,88]. Since it is well established that cis isomers absorb light around 330 nm, some inner filter effects may arise from such cis isomers, which may somewhat increase the importance of this mechanism of protection and cis carotenoids quench ^1^O_2_ less efficiently than all-trans isomers [86], so the ^1^O_2_ quenching mechanism will be decreased.

### 4.5. Singlet Oxygen—Cancers

While ^1^O_2_ can be generated non-photochemically [82,83], its generation via light in the skin and the eyes is of most importance. The possibility that dietary carotenoids, especially β-carotene, might reduce human cancers, including those in the skin due to UV/visible radiation and ROS, including singlet oxygen, has been discussed for many years, (see, for example, [107]) with contradictory claims both with respect to its effectiveness and its safety. As noted above, the results from the ATBC and CARET clinical trials of β-carotene and lung cancer, along with experimental results from carotenoids and UV-carcinogenesis, have raised concerns regarding the safety of β-carotene supplementation and the warning that it should not be used in cancer prevention in the general population [1,81]. However, as summarized by Bayerl [58] and Góralczyk [108], the methodology of these studies has been questioned. There seems no doubt that the use of β-carotene to treat EPP and other photosensitive diseases is worthwhile, but clearly smoking should be strongly discouraged for such people. Furthermore, this review suggests short term use (e.g., for vacations in regions with high UV exposure), particularly for non-smokers, would be worth considering.

There are also differences in the claims for β-carotene benefits against squamous cell carcinoma, ranging from inhibition of the proliferation of these cells [109] to no evidence being found that carotenoids play an important protective role against incident squamous cell carcinoma of the skin [110]. However, there is some degree of agreement that a combination of carotenoids with other antioxidants, including vitamin E and C, are likely to offer some skin protection [16,111,112].

Several other carotenoids have been discussed as possible beneficial antioxidants in the skin, including lycopene, astaxanthin, lutein and, to some extent, such benefits for the skin seem likely [113,114]. Perhaps the most exciting claims concern zeaxanthin and skin melanomas [115] and earlier studies with fucoxanthin. However, these findings do not involve ^1^O_2_ and are out of the scope of this review.

The overall situation is summarized in a recent review [113]. As these researchers point out, although UV radiation is a main factor in the development of non-melanoma skin cancer and melanoma, studies do not, for certain, confirm the protective role of carotenoids in skin photocarcinogenesis.

In the eye, there is little or no involvement of carotenoids and ^1^O_2_ with regard to the development of cancer. However, there are claims, as noted above, that zeaxanthin (which can significantly help against AMD, see below) may also have anti-carcinogenic properties which have been studied for the treatment of various cancers. These do not appear to include melanoma of the eye [116]. However, one speculation [117] does concern ^1^O_2_ and both eu- and phaeomelanin. Both these melanins bind transition metals and, hence, can lead to radicals via Fenton reactions. The melanins associate with the minor grooves in DNA which allow them to both damage DNA and reduce access to DNA repair enzymes. However, melanin can react with ^1^O_2_ [118], possibly leading to structural modifications in the melanin itself and, hence, affecting the role of the transition metals redox reactions. This work suggests a dual role for melanins as pro- and antioxidants and may offer improved strategies for using PDT to treat melanoma. In a somewhat related study [119], models of both melanins have been shown to react with both oxidizing and reducing radicals.

### 4.6. Singlet Oxygen—Age-Related Macular Degeneration (AMD)

AMD is a major cause of loss of sight in the Western world. Of the over 25 or so carotenoids in human serum only lutein and zeaxanthin accumulate in the macula (zeaxanthin is in two stereo isomeric forms, one of which is called meso-zeaxanthin—the structures are given in Figure 3). AMD may well be related to reduced levels of lutein and zeaxanthin (macular pigment) in the diet, serum or retina, and blue light exposure. However, several groups claim that lycopene also has a role in reducing AMD even though there is no lycopene in the macula [120,121,122]; a possible molecular mechanism, based on radical reactions, is discussed below.

Somewhat related to the mechanisms associated with porphyria, at least two roles for the macular pigments are now accepted [123]—a simple filter effect and an antioxidant effect to minimize the damage due to ^1^O_2_ and other ROS. As shown in Table 3 above, the efficiency of quenching ^1^O_2_ is about double for zeaxanthin and meso-zeaxanthin than lutein in benzene. Furthermore, in liposomes, the quenching rate constants for zeaxanthin and lutein show a similar ratio—2.3 and 1.1 × 10^8^ dm^3^ mol^−1^ s^−1^, respectively. These results possibly suggest that a major role of zeaxanthin in the macula is ^1^O_2_ quenching, but that such quenching may be a less important function of lutein [90]. However, as the concentration of the carotenoid increases (in the liposomes) there are significant differences between lutein and zeaxanthin. The lutein quenches the ^1^O_2_ in a linear manner at all concentrations used (up to 70 μM), while the quenching by zeaxanthin starts to become less efficient above about 30 μM. Indeed, at 70 μM zeaxanthin shows no ^1^O_2_ quenching at all under these conditions when the ^1^O_2_ is generated in the aqueous phase (using rose bengal, a water-soluble sensitizer to generate the ^1^O_2_). When the ^1^O_2_ is generated within the liposome, the loss of quenching efficiency is less marked, but is still evident above about 40 μM. Since the macular pigment concentrations are relatively high, this may suggest ^1^O_2_ quenching is important for lutein and that the results in simple organic solvents such as benzene do not account for this aggregation effect.

It is noteworthy that the efficiency of ^1^O_2_ quenching by other dietary carotenoids in liposomes are much higher [90] (e.g., β-carotene = 24 × 10^8^ dm^3^ mol^−1^ s^−1^) and, of course, there is much more β-carotene in our diet than any of the macular pigments. Thus, the reasons for the specific carotenoid pigments accumulated in the macula are far from just that of efficient ^1^O_2_ quenchers. Overall, the possible role of the different carotenoids with respect to the efficiency of ^1^O_2_ in the macula is still not totally understood.

### 4.7. Radicals—General (In Vitro)

As noted above, epidemiological studies have linked the intake of carotenoids with the reduced risk of degenerative diseases. However, trials using dietary carotenoid supplements have led to the possibility that damaging pro-oxidant reactivity can also arise. Likely reasons for such a switch in behavior include the properties of the carotenoid radicals themselves.

Carotenoid interaction with oxidizing radicals (and, to a lesser extent, with reducing radicals) is far more complex than with ^1^O_2_. This is because several quite different radicals are important and several types of reaction, with oxidizing radicals, can occur. For example, the hydroxyl radical (OH•) and some sulfur-based radicals add to carotenoids to give a neutral adduct radical, while nitrogen dioxide (NO_2_•) and thionyl (RSO_2_•) radicals can oxidize a carotenoid to its corresponding radical cation and the superoxide radical anion (O_2_•^−^) is generally regarded as unreactive. The corresponding conjugate acid, HO_2_•, is much more reactive than O_2_•^−^ (O_2_•^−^/HO_2_• pKa is 4.7) and reactivity for O_2_•^−^ at pH values near neutrality could be due to the small amounts of HO_2_•, present even at such pH values. Theoretical studies, not reviewed here, claim carotenoids may undergo an electron transfer with O_2_•^−^ to yield the corresponding carotenoid radical anion—this is quite different from the oxidation of carotenoids by a number of other oxidizing free radicals [124].

Neutral carotenoid radicals, including adducts, are not easy to study, both because their spectra often overlap with the intense ground state absorption and because there are several possible neutral radicals—see below. A further complexity arises because, once formed, neutral radicals can add molecular oxygen, forming peroxyl radicals, and this can lead to a switch from the antioxidant to pro-oxidant behavior of carotenoids. This has been observed for β-carotene in a non-biological environment [76] and, more recently, for lycopene in human cells [125]. Such anti-/pro-oxidant roles of carotenoids and xanthophylls are of particular interest to photosynthesis, vision and cancer.

### 4.8. Radicals—Simple Solvents, Micelles and Liposomes

Early work, using pulse radiolysis [126], subsequently [127,128] established the spectra of the carotenoid radical cations (CAR•^+^). These radicals can also be generated in other ways, such as by flash photolysis [129,130,131], electrochemically [132,133] and chemically [134]. Pulse radiolysis was also used to generate and characterize the radical anions of the carotenoids [126,135].

These techniques have led to the measurement of the one-electron oxidation potentials of a number of carotenoids to be measured for aqueous micellar solutions—they are all near 1000 mV, which means carotenoid radical cations are quite strong oxidizing agents themselves [127,128,136,137]. Thus, carotenoid radical cations may oxidize important biomolecules, such as tyrosine, tryptophan and cysteine. Moreover, electron transfer between many pairs of carotenoids have been observed. This allowed the relative one-electron oxidation potentials of several carotenoids to be obtained. Such studies demonstrated that lycopene has the lowest potential, i.e., it is the most easily oxidized of the carotenoids. A consequence of this could be that lycopene is the “sacrificial” carotenoid in vivo, when there is a mixture of carotenoids present, and this may be related to health benefits often reported for dietary lycopene—see, for example, [122,138].

The high-energy fast reaction technique of pulse radiolysis has also demonstrated that water-soluble antioxidants, such as vitamin C, can convert (water-insoluble) carotenoid radical cations back to the parent carotenoid [137]. Thus, a potentially deleterious pro-oxidant effect due to the oxidizing potential of carotenoid radical cations may be reduced by ascorbic acid. It is also established that the free radicals in cigarette smoke will reach the lungs. For heavy smokers, it has been shown that a high concentration of dietary β-carotene can have a damaging effect, and a possibility is that this may be related, in part, to smoke-based free radicals (e.g., NO_2_•) reacting with β-carotene to produce the β-carotene radical cation, which can then damage bio-substrates. A related and interesting observation, with respect to the mechanisms of photosynthesis, has come from work reported by Skibsted et al. [130,131]. These studies used pH conditions where the standard reduction potentials were the same for tyrosine and tryptophan. They found that tyrosine reacted with the carotenoid radical cation an order of magnitude faster than tryptophan and suggest that this may be related to tyrosine, rather than tryptophan, being the protein moiety reacting with β-carotene in the protective mechanism, which operates in the photosynthetic reaction center. These researchers also point out that the driving force in such processes depends on the “local” pH, so the reverse reaction between a tyrosine radical and β-carotene may also be important in proteins.

Finally, more than one neutral radical can arise from various routes:

HCAR•^+^ → CAR• + H^+^ (1)

(note: here, we have abbreviated the radical cation as HCAR•^+^ rather than CAR•^+^ to illustrate the deprotonation),

HCAR = O•^−^ + H_2_O → HCAR-OH• + OH^−^ (2)

where the radical anion of a carbonyl containing carotenoid reacts with water to give a neutral adduct, and:

CAR + RS• → CAR-RS• and CAR + OH• → CAR-OH•(3)

Carotenoid radicals have also been generated and studied in micelles and liposomal cell models. These reactions and subsequent processes have been reviewed recently [139].

Carbon tetrachloride is known to cause various types of tissue injury and, using trichloromethylperoxyl radical (CCl_3_O_2_•) as a model of alkylperoxyl radicals, and Triton X micelles as a cell membrane model, Hill and co-workers [140] reported that pulse radiolysis gave two distinct species, a rapidly formed carotenoid radical cation and a species assigned to an addition product, the addition product decaying to give a more radical cation, i.e., the CAR•^+^ is formed via the fast:

CCl_3_O_2_• → CAR•^+^ + CCl_3_O_2_•^−^ , (4)

and slower:

CAR-CCl_3_O_2_• → CAR•^+^ + CCl_3_O_2_•^−^ (5)

(with astaxanthin, only the slow process was observed).

Results from comparing the detergent and totally non-polar environments (benzene) suggest there is more than one distinct environment for β-carotene in these micellar cell membrane models. Indeed, if the TX 100 is a useful cell model, this suggests such a range of ‘sites’ may well have biological consequences.

### 4.9. Radicals—Ex Vivo Cell Studies

Boehm and co-workers [125] have reported the significant effect of the concentration of oxygen on the protection of human lymphoid cells against high-energy γ-radiation by lycopene. In this study, volunteers either took a large lycopene diet or near-zero lycopene and their lymphoid cells were irradiated with γ-radiation from a ^60^Co source. This radiation leads mainly to the production of two reactive radical species:

H_2_O → OH• + e_aq_^−^ (6)

(plus a small amount of H• and non-radical species).

In the presence of nitrous oxide (N_2_O), the solvated electrons are converted to more OH• and, with oxygen-flushed formate solutions, virtually only O_2_•^−^ is generated [141].

These researchers used standard cell-staining techniques with eosin and measured membrane destruction and cell death. Using γ-radiation doses up to 5000 Gy (and normal atmospheric conditions), the dietary lycopene was found to protect the cells four to five times more efficiently compared to a virtually zero lycopene diet. Interestingly, a really substantial effect of oxygen concentration was also reported. Thus, at near zero concentrations of oxygen the cells were totally protected by the lycopene, while at 100% oxygen there was no protection at all by the lycopene [125]. A similar effect was seen for β-carotene with more or less the same protection from zero to 20% oxygen as for lycopene (to be published). At very high oxygen concentrations (above 60%), the β-carotene was a more efficient protector than lycopene. Moreover, in preliminary studies, the macular pigments, zeaxanthin and lutein, were shown to offer more protection as the oxygen concentration increases—far better than for either lycopene or β-carotene (to be published). These researchers compared this ‘oxygen’ effect to the earlier observations of Burton and Ingold [75] who used non–biological conditions. A proposed mechanism involved cell protection via quenching of the reactive OH• by lycopene (either by hydrogen abstraction or addition) then the formation of a reactive peroxyl neutral radical via oxygen addition. This peroxyl radical was proposed as the major species leading to the cell membrane destruction. Whatever the mechanism (e.g., involving neutral lycopene radicals and oxygen addition) this, substantial difference, in cell protection, due to oxygen concentration, may (with appropriate methods) be useful clinically by leading to a mitigation of damage caused by radiation treatment of tumors. From a simplistic point of view (after a high lycopene diet), saturating the tumor with oxygen should cause no effect on the radiation therapy benefit, while flushing the non-necrotic areas with, say, nitrogen could protect against the unwanted radiation damage. This oxygen addition mechanism may also offer another explanation for the (lung) damaging effect of high doses of β-carotene in heavy smokers discussed above. If radicals, such as NO_2_•, present in cigarette smoke can also add to β-carotene (as well as oxidize it as described above), then the high oxygen concentration in the lungs could increase the possibility of oxygen addition to the initial neutral radical adducts, leading to a damaging carotenoid peroxyl radical. For NO_2_• itself, the adduct (if formed) is short-lived, so this may not be an important process, but, of course, it could be important for other radicals present in cigarette smoke. We have previously noted vitamin C can convert damaging carotenoid radical cations (which can result from oxidation by, for example, NO_2_•) back to the parent carotenoid, but there is no information on a corresponding process with carotenoid peroxyl radicals.

The radiation protection study described above [125] allowed a comparison of the effects of the radicals generated on cell kill. Under typical conditions, a cell death of 5.60%, 8,75% and ≤ 2.18% was detected for OH•, O_2_•^−^ and e_aq_^−^, respectively, which indicated that solvated electrons are inefficient at inducing cell kill, whilst superoxide radical is the most effective radical at causing cell death.

Furthermore, as noted above, other dietary antioxidants may interact with free radicals and this can lead to enhanced protection. However, in this study, no such enhancement was observed for the cell protection by lycopene, with the addition of vitamins C and E. This implies that lycopene radical cation is not produced during the process of lycopene protection against γ-radiation.

As noted in [125], metal ions may also be involved in cell damage via Fenton reactions [142], but these are not involved in the above study.

A wide range of other ex vivo studies on skin models, atherosclerosis, AMD and cancers have been reported previously [84,139].

### 4.10. Radicals—The Skin and EPP

It is generally accepted that EPP involves light absorption by PPIX, leading to the PPIX triplet state followed by energy transfer to oxygen to generate ^1^O_2_, which then damages the skin. The protective mechanism of β-carotene is believed to be based on the efficient quenching of the ^1^O_2_ by the β-carotene.

However, as discussed above, Ogilby and co-workers [101] have been unable to detect singlet oxygen in a single cell, laser-based system. Thus, other protective processes involving β-carotene should be considered. One such non-radical process is direct quenching of the PPIX triplet state by β-carotene. This leads to the triplet of the quencher (β-carotene) which then dissipates the energy as heat. This seems somewhat unlikely due to the mobility of the oxygen. However, it must be remembered that the quenching of the PPIX triplet by normal (ground state triplet oxygen) is nearly an order of magnitude less than the diffusion-controlled rate constant (i.e., only about one ninth of the collisions between the oxygen and the triplet PPIX will result in ^1^O_2_).

Perhaps more likely is the formation of a range of free radicals due to UVA/UVB incident on the skin, which leads to porphyrin radicals which, in turn, can be quenched by the β-carotene. However, the generally accepted mechanism of ^1^O_2_ generation from the PPIX triplet seems more likely at this time.

### 4.11. Radicals—Eye Cancers and AMD

UVA and long wavelength UVB radiation is virtually completely filtered out before it reaches the macula. However, there are a variety of endogenous photosensitizers within the retina which can be excited by the light that does reach it. The outer retina (containing the photoreceptors and retinal pigment epithelium (RPE)), is immediately adjacent to the choroidal blood supply and, as such, is highly oxygenated. Therefore, favorable conditions for photodynamic damage arise. The fact that there is a strong dependence of oxygen concentration on the susceptibility of the retina to photodamage suggests that light-induced damage to the retina is indeed due to the photodynamic processes leading to singlet oxygen production, and is related to AMD and not cancers. However, other parts of the eye have much less light/UV filtering and this can lead to ocular melanoma. Eye melanoma most often affects the middle layer of the eye (uvea), the colored portion of the eye (iris), the muscle fibers around the eye’s lens (ciliary body), and the layer of blood vessels that line the back of the eye (choroid). The iris is exposed to both visible and UV radiation, even though some of the UV is removed by the cornea, with the precise amount depending, of course, on the specific wavelength. This light exposure can lead to iris melanoma. The iris contains various pigments including melanins and haemoglobin. The melanin is reduced upon aging, typically by about half at 65 years of age. It is well established that the melanin has a major protective role via sequestering metal ions, and light absorption. There are two major classes of melanin, black–brown eumelanin and yellow–red sulfur-containing phaeomelanin. Both types of melanin can be free radical quenchers for both oxidizing and reducing radicals [143].

While the biological relevance of this ability to quench free radicals is not fully established, these results do show that melanins may act as a protector against free radical damage, including cancers, by this additional protective role. However, interactions with dietary carotenoids are less well established. The iris, ciliary body, and RPE/choroid (the uveal structures) contain about 30% of the eye’s lutein and zeaxanthin, plus other carotenoids. As noted above, when carotenoids react with some oxidizing radicals, such as NO_2_•, the carotenoid radical cation is formed as a consequence of the removal of the damaging NO_2_•. Since, as shown above, such radical cations are rather strongly oxidizing themselves, this can lead to a switch to pro-oxidative damage rather than anti-oxidative protection. However, Edge and co-workers [141] have shown that both types of melanin can react with carotenoid radical cations (including lutein and zeaxanthin) and reduce them back to the parent carotenoid. The melanins act as a free radical ‘sink’, but the reactivity of melanins with carotenoid radical cations may be an additional role for melanins in protecting the in vivo system from oxidative damage by carotenoid radical cations. Moreover, where the concentration of carotenoids are low but pivotal to the protective processes, this behavior of the melanins may help to maintain the concentration of the key carotenoids, such as lutein and zeaxanthin.

A surprising observation concerns a role for lycopene in protecting against AMD [120,122]. We suggest the most likely mechanism for such a protection is based in the relative redox properties of lycopene compared to lutein and zeaxanthin.

**Figure 6:**
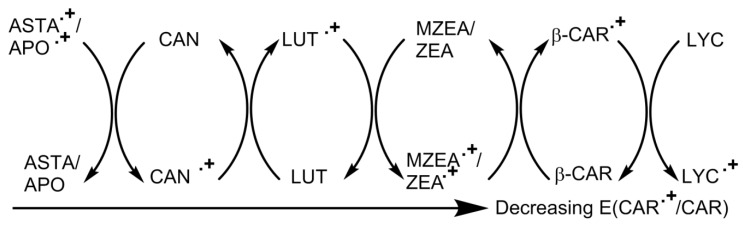
Scheme showing the relative ordering of the one-electron reduction potentials of several carotenoid radical cations in benzene.

Using pulse radiolysis, we have shown the order of such potentials, as shown in Figure 6 [136]. Thus, it seems possible, that lycopene, even though it is not found in the macula, can help to preserve the lutein and zeaxanthin en route to the macular. If this is true it would suggest that protection against AMD should not just be linked to lutein and zeaxanthin supplementation, but also to a diet rich in tomato lycopene.

### 4.12. Radicals—Cancer

The benefits and problems associated with carotenoids and cancer are summarized above. Despite many in vitro and ex vitro studies indicating the antioxidant benefits of carotenoids against many cancers via free radical quenching, there is little or no certainty that carotenoids reduce the incidence of cancer by such a simple mechanism. As well as such possible antioxidant benefits, carotenoids may also influence several aspects of carcinogenesis. A wide range of cancers and the possible benefits of carotenoids have been reviewed by Rock [144].

As described above, animal studies have compared closed-formula and semi-defined diets with β-carotene, with some work reported on lycopene and astaxanthin. Understanding the molecular mechanisms associated with β-carotene-fed animal studies and UV radiation is problematical for several reasons. Firstly, there is a significant difference between the so-called closed-formula diet and the semi-defined diets (see above). The ‘closed formula’ diet can vary from experiment to experiment and show the photoprotective effect of the carotenoid. However, the semi-defined diet (where, amongst other factors, the vitamin supplements are defined) show not only no protection of β-carotene against UV-induced tumors, but also a significant exacerbation. This effect, for the semi-defined diet, was also observed with β-carotene replaced by astaxanthin, but not by lycopene. Another factor not always discussed in such work (and other studies using UV radiation) is that the UV will produce not just a single oxidizing radical, but several radicals so that the damage can result from more than one mechanism.

Moreover, important in explaining the different responses caused by β-carotene is the finding that β-carotene shows efficient radical trapping antioxidant behavior at normal oxygen concentrations (i.e., normal partial pressure), while at higher oxygen concentrations, the carotenoid changes from being an antioxidant and exhibits pro-oxidant properties [75,145]. Thus, β-carotene has been shown to have either limited antioxidant protection or to be a pro-oxidant under oxidative stress conditions [146]. The carotenoid reacts efficiently with peroxyl radicals (RO_2_•) where the reaction (electron transfer) produces the carotenoid radical cation (CAR•^+^), as shown in Equation (7) [147].

RO_2_• + β-CAR → RO_2_^−^ + β- CAR•^+^ (7)

Whether the physiological environment, e.g., oxidative stress, can modulate the likelihood of this reaction is uncertain. However, as described above, carotenoid radical cations are quite strong oxidizing species, with a reduction potential of about 1000 mV [127,128,147]. Such radicals, if formed, could contribute both directly to membrane damage, and indirectly to the pro-oxidant capacity of the ‘parent’ carotenoid. This may well be related to whether the carotenoid radical cation could be repaired. Based on electron transfer rate constants between various carotenoids and their interactions with vitamin E (α-tocopherol, (TOH) and vitamin C (AscH¯), both components of animal rations, a mechanism was suggested for the interaction of β-carotene that would lead the repair of the β-carotene radical cation (Equations (8)–(10)) [136,137].

RO_2_• + TOH → TOH•^+^ + ROOH (8)

TOH•^+^ + β-CAR → TOH + β-CAR•^+^ (9)

β-CAR•^+^ + AscH ¯ → β-CAR + Asc•− + H^+^ (10)

Such a mechanism suggests vitamin E reacting with an oxy-radical to form the vitamin E radical cation and β-carotene repairing this radical cation to form the β-carotene radical cation (β- CAR•^+^), as in (9). This, in turn, could be repaired by vitamin C as in (10). That is, β-carotene would not only protect by directly quenching the reactive oxy-radical, as shown in Equation (7), but also enhance the radical protective properties of both vitamins E and C, shown in Equations (8)–(10). Of course, UV can produce many other radicals and oxidizing radicals, e.g., (RO_2_•), and these may also react with β-carotene to produce the carotenoid radical cation directly.

Overall, these animal dietary studies do not support a proposed scheme, as suggested by Equation (10), of the β-carotene radical cation being repaired by ascorbic acid. If the mechanism does not apply, then the reduced intermediates, in the case of the β-carotene radical cation, behave as pro-oxidants. While the mechanism may remain uncertain, it is obvious that dietary factors can have a significant effect on the capacity of β-carotene to influence carcinogenic expression [74]. These factors may, of course, be other carotenoids, their isomers, or some yet unidentified phytochemical (Equation (11)).

β-CAR•^+^ + ? → β-CAR + H^+^ + ? (11)

The ex vivo results, discussed above (Section 4.9) for β-carotene and other carotenoids, may suggest that an important factor is molecular oxygen. Once a neutral radical such as OH• has formed a neutral adduct with β-carotene (to give HO-Carotene•), an oxygen molecule can be added to give a damaging peroxyl radical (HO-Carotene-OO•), leading to enhanced cell membrane damage and, hence, possibly to increased cancer risk.

It has been suggested that β-carotene, (which is so prevalent in the green and yellow vegetables and fruits, and which led to the proposal that the carotenoid could reduce cancer risk), merely cloaks the real anti-cancer agent [80] The results from the ATBC and CARET clinical trials of β-carotene and lung cancer, along with the experimental results with the carotenoid and UV-carcinogenesis, have raised concerns regarding the safety of β-carotene supplementation and the warning that it should not be used in cancer prevention in the general population [1,81].

**Figure 7:**
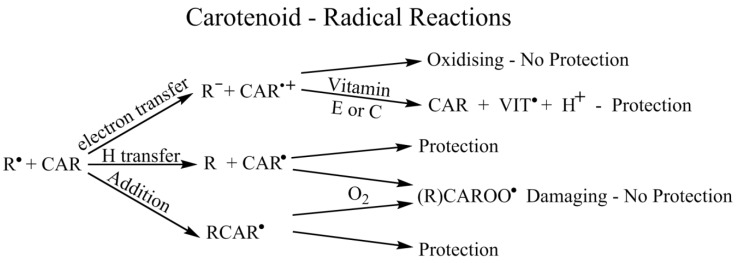
Summary scheme of carotenoid reactions with free radicals.

The various molecular mechanisms for radicals and carotenoids can be summarized in the following scheme (Figure 7):

## 5. Summary

Carotenoids are widespread in nature and in commercial use. From photosynthesis to porphyria and from cancer to commercial food dyes, they play varied and pivotal roles. Indeed, in photosynthesis, they both protect the system from too much light and act as auxiliary pigments when the light is insufficient for efficient photosynthesis. Apart from photosynthesis, the carotenoids’ major roles in human health are as protective pigments and, for β-carotene, precursors for essential vitamins and metabolites that are requisites for good health. In the commercial world, their main use is as “natural” colorants - both directly, as additives to our foods, and indirectly via feeding materials for, for example, chickens and salmon.

However, the protective function of certain carotenoids, particularly β-carotene and lycopene, has been challenged as a result of clinical trials where β-carotene supplementation in male smokers resulted in significantly increased incidence of lung cancer, and from animal studies in which β-carotene supplementation resulted in the exacerbation of UV-carcinogenic expression. Indeed, dietary studies have shown diet influences the carcinogenic response to β-carotene and that β-carotene, per se, is pro-carcinogenic in UV-carcinogenesis. These findings challenge the use of β-carotene as an anti-cancer agent for the general population.

If we are to understand the contradictory reports of beneficial associations and detrimental effects in clinical trials and experimental animal studies, we must first understand the molecular mechanisms involving singlet oxygen and antioxidant reactions in which carotenoids are involved. Furthermore, we must understand the individual properties of the carotenoid in participating in such reactions and recognize that some carotenoids, e.g., β-carotene, may act as a pro-oxidant under specific conditions. Thus, the introduction of an antioxidant carotenoid into the complex milieu of a cell can result in unexpected consequences.

Among the molecular mechanisms, singlet oxygen is a highly damaging species and dietary carotenoids directly quench the source of singlet oxygen-photosensitizer triplets, such as those of porphyrins and chlorophylls. A significant quenching of singlet oxygen itself, of a magnitude observed in organic solvents, was also reported from human lymphocytes and water-soluble β-carotene. Although slightly lower, similar quenching was observed with oral intake of β-carotene at concentrations used in everyday supplementation. Lycopene, from the diet, also provided a significant protection factor for singlet oxygen quenching. Nevertheless, the ability of β-carotene to quench this oxygen species has been questioned after studies where individual cells reported no such quenching. On the other hand, it has been demonstrated that β-carotene does quench some singlet oxygen in isolated PSII II. Singlet oxygen quenching by β-carotene is a major process in the protection mechanism in porphyria. Related mechanisms associated with porphyria are now accepted for AMD. One mechanism is that the carotenoids exhibit antioxidant effects that minimize singlet oxygen quenching and other ROS. The generation of singlet oxygen by UV/visible light in the skin is of importance and is a primary reason for the speculation that β-carotene might reduce cancer risk. However, the role of β-carotene quenching of singlet oxygen in the etiology of skin cancer has been controversial. One major observation from all of these studies, particularly those of PSII, is that the proximity of the carotenoid to the singlet oxygen source is a major consideration for quenching to occur.

Pulse radiolysis was used to establish the spectra of carotenoid radical cations and radical anions. This technique also allowed the one-electron oxidation potentials of several carotenoids to be measured in aqueous micellar solution. The radical cations typically exhibited an oxidation potential of near 1000 mV, indicating that they are strong oxidizing agents. It has been suggested that the β-carotene radical cation could be involved in the damaging effects to lungs of heavy smokers, particularly those with low levels of ascorbic acid that have the potential of reducing the carotenoid radical cation. Such a mechanism has been proposed for the pro-carcinogenic activity of β-carotene in UV-carcinogenesis, although, in the latter, dietary studies have shown that vitamin C level has no potentiating effect on β-carotene exacerbation on UV-carcinogenesis. It must be remembered that mice are able to synthesize their own vitamin C requirement and the proposed mechanism for lung damage in humans should still be considered as a serious proposal; however, it would be much stronger if the carotenoid radical cation could be identified in the target tissue.

Aware of the unique property of β-carotene and lycopene to “switch” from antioxidant to pro-oxidant under varying oxygen concentrations, investigators have reported a substantial effect of oxygen concentration on the protection of human cells against ϒ-radiation by lycopene. At very high oxygen concentrations, β-carotene was a more efficient protector than lycopene. ϒ-radiation leads, mainly, to two reactive radical species—hydroxyl radical and superoxide anion. At zero-oxygen concentration, cells containing lycopene exhibited virtually no damage due to the high-energy radiation, whereas lycopene gave no protection at all at 100% oxygen concentration. Furthermore, no enhancement with supplemental antioxidants, vitamins C and E, occurred, indicating that the lycopene radical cation was not involved in the γ-radiation protection mechanism and it is likely to involve addition processes with both a (neutral) radical and oxygen, giving damaging peroxyl radicals. These observations may have important clinical potential for protecting normal tissue when exposed to high radiation levels during the treatment of affected target cells.

There was formidable epidemiological evidence, before the β-carotene intervention trials were conducted, that the carotenoid would exhibit anti-cancer properties. Indeed, β-carotene was known to be a strong singlet oxygen quencher and antioxidant, and this formed the basis for the enthusiasm that the carotenoid would act as an anti-cancer agent. However, the study of the molecular mechanism(s) of β-carotene has revealed knowledge of the carotenoid and its chemical properties under various microenvironments that clearly demonstrate that the carotenoid’s interactions are complex and must be taken into account to explain the volumes of research and clinical results observed. The fact that it can act as antioxidant or pro-oxidant, the fact that the carotenoid can form radical ions that themselves may participate in cellular damage, the conditions under which β-carotene acts as an efficient singlet oxygen quencher and knowledge of the conditions in which the carotenoid is introduced are all conditions to be considered before declaring its biological response. It is only through continued study of the “antioxidant” itself that we will be able to assess the potential beneficial and/or harmful effects of the respective carotenoids. Whereas a diet rich in carotenoid-containing fruits and vegetables may be beneficial in the fight against some diseases, one must remain cognizant of the potential risks that carotenoid supplementation may pose.

## Figures and Tables

**Table 1:** Influence of diet on β-carotene-mediated UV-carcinogenic expression*.

Diet	Median Tumor Time, Weeks	Tumors/Animal
**Closed formula**		
Control	20.6	0.52
0.07% β-Carotene	20.0	0.60
**Semi-defined**		
Control	19.5	0.60
0.07% β-Carotene	17.2	1.63

* There was no significant influence of β-carotene on tumor latent period (median time to tumor) or tumor multiplicity (mean number of tumors/animal) in animals fed the closed-formula diet. Nor were there differences in either tumor parameter between closed-formula and semi-defined control diets. However, β-carotene supplementation of the semi-defined ration significantly shortened the tumor latent period (*p* < 0.002) and increased tumors (*p* < 0.03) [74].

**Table 2:** Influence of varying levels of vitamins C and E on β-carotene-modulated tumor multiplicity (average number of tumors per animal at median tumor time).

Control (−βCar) ^a^	(+βCar) ^b^	(+βCar, −Vit C) ^c^	(+βCar, −Vit C, low Vit E) ^d^
1.05	3.20	3.45	5.90

^a^ Control, semi-defined diet containing 110 mg vitamin E and 990 mg vitamin C/kg of diet; ^b^ (+βCar): semi-defined diet containing 0.79g/kg diet of β-carotene with equivalent levels of vitamins C and E as Control; ^c^ (+ βcar, −Vit C): same composition as + βCar but with no vitamin C; ^d^ (+βCar, −Vit C, low Vit E): same composition as +βCar, −Vit C but with only 49 mg/kg diet of vitamin E, the same as in closed-formula ration [79]. (Reprinted by permission of the publisher Taylor and Francis, Ltd., http://tandfonline.com).

**Table 3:** Second-order rate constants (k_q_) for the carotenoid quenching of ^1^O_2_ in benzene.

Carotenoid	n	k_q_/10^9^ dm^3^mol^−1^s^−1^
lycopene	11	17.0
β-carotene	11	13.0
zeaxanthin	11	12.6
*Meso*-zeaxanthin	11	12.2
α-carotene	10	12.0
lutein	10	6.64
septapreno-β-carotene	9	1.38
